# Oral tolerance and functional Treg cells are induced in BALB/c mice after gavage with bovine β-lactoglobulin (BLG)

**DOI:** 10.1186/2045-7022-1-S1-P37

**Published:** 2011-08-12

**Authors:** Karine Adel-Patient, Hervé Bernard, Sophie Wavrin, Sandrine Ah-Leung, Jean-Michel Wal

**Affiliations:** 1 INRA, Unité d'Immuno-Allergie Alimentaire, Gif-sur-Yvette, France

## Background

Food allergy is considered as resulting from an impaired development of oral tolerance, or a breakdown in existing oral tolerance. We then aimed to induce oral tolerance to a major cow's milk allergen, BLG, which could prevent further systemic allergic sensitization and elicitation. Mechanisms involved were investigated.

## Methods

BALB/cJ mice were gavaged with PBS (control mice) or with 2 mg of purified BLG on days 1, 2, 3, 8, 9 and 10. All mice were then sensitized by i.p. administration of 5 µg of BLG in alum on day 14. Mice sensitization was assessed by quantitative measurement of BLG-specific IgE and IgG1 antibodies on individual serum samples collected on day 36. After a boost administration of BLG/alum, elicitation of the allergic reaction was induced by intra-nasal administration of BLG. Bronchoalveolar lavage fluids (BAL) were collected 24h later and their cellular composition was analysed using simultaneous labelling with anti-CD3, anti-B220, anti-CMHII, anti-CD11c and anti-CCR3 or with anti-CD4 and anti-Foxp3 antibodies. In parallel, Th1/Th2/Th17 cytokines were assayed on centrifuged BAL.

## Results

Both allergic sensitization and elicitation were efficiently induced in control mice, as demonstrated by the high levels of anti-BLG IgE and IgG1 antibodies in sera, and IL-4, IL-5, GM-CSF and eotaxin release and eosinophil influx in BAL. Conversely, BLG-specific IgE and IgG1 antibody productions, as well as cytokine secretion and eosinophil recruitment in BAL, were totally inhibited in mice gavaged with BLG before sensitization. Interestingly, a high percentage of Foxp3+ cells within CD4+ cell population and a negative correlation between the number of eosinophils and the percentage of Foxp3+ cells were evidenced in BAL of mice gavaged with BLG.

## Conclusion

Both inhibition of the allergic sensitization and active suppression of effector cells by Foxp3+ cells at the challenging site may contribute to the efficient systemic tolerance in BALB/c mice after gavage with BLG.

